# Transplant Trial Watch

**DOI:** 10.3389/ti.2025.14603

**Published:** 2025-03-26

**Authors:** John O’Callaghan, John Fallon

**Affiliations:** ^1^ Centre for Evidence in Transplantation, Nuffield Department of Surgical Sciences, University of Oxford, Oxford, United Kingdom; ^2^ University Hospitals Coventry & Warwickshire, Coventry, United Kingdom

**Keywords:** randomised controlled trial, liver transplantation, lung transplantation, extracorporeal photopheresis, survival

To keep the transplantation community informed about recently published level 1 evidence in organ transplantation ESOT and the Centre for Evidence in Transplantation have developed the Transplant Trial Watch. The Transplant Trial Watch is a monthly overview of 10 new randomised controlled trials (RCTs) and systematic reviews. This page of Transplant International offers commentaries on methodological issues and clinical implications on two articles of particular interest from the CET Transplant Trial Watch monthly selection. For all high quality evidence in solid organ transplantation, visit the Transplant Library: www.transplantlibrary.com.

**Randomised Controlled Trial 1**

Multicenter Randomised Controlled Trial of Single Versus Double Venous Outflow Reconstruction in Right Lobe Living Donor Liver Transplantation- Venous Outflow in Liver Transplantation (VOLT) Trial.

*by Reddy, M. S., et al. Annals of Surgery 2024 [record in progress].*

## Aims

The authors aim to compare early patency of the reconstructed anterior sector vein (neoMHV) and clinical outcomes between a single outflow technique (SOT) and double outflow technique (DOT) in right lobe living donor liver transplantation (RtLDLT).

## Interventions

One arm received the double outflow technique (DOT): Separate anastomoses of the right hepatic vein (RHV) and the prosthetic neo-middle hepatic vein (neoMHV) to the recipient inferior vena cava (two openings). The other arm received the single outflow technique (SOT): Conjoint venoplasty on the back table, creating a single common outflow orifice (RHV + neoMHV together) that is then anastomosed *en bloc* to the recipient vena cava (one opening).

## Participants

219 adult patients undergoing right lobe LDLT who required prosthetic anterior sector vein (ASV) reconstruction. To be included grafts needing at least one reconstructed ASV (>4 mm). Prosthetic grafts (PTFE or Dacron) used. Key exclusion criteria were: retransplant, graft with middle hepatic vein included, non-prosthetic reconstructions, contraindication to contrast imaging.

## Outcomes

The primary outcome was NeoMHV (anterior sector vein) patency at multiple time points (2, 4, and 6 weeks post-transplant) evaluated by Doppler ultrasound and cross-sectional imaging. The secondary outcomes were: Intraoperative metrics (cold ischemia time, graft implantation time, blood loss, etc.), postoperative complications (e.g., vascular/biliary events, Clavien-Dindo classification, Comprehensive Complications Index), early allograft dysfunction, ICU/hospital length of stay & In-hospital, 90-day, and 1-year patient survival.

## Follow-Up

Primary patency assessments up to 6 weeks post-transplant. Additional postoperative outcomes (including survival) tracked up to 1 year (median survival data reported).

## CET Conclusion

*by John Fallon*

The authors conducted a well-designed and blinded Multicentre, randomised controlled trial at 5 LDLT centres in India. 219 recipients were included in the study with 110 undergoing SOT and 109 DOT. They demonstrated NeoMHV Patency was significantly better at 2 weeks (92.5% vs. 82.9%, p = 0.032) and 4 weeks (84% vs. 69%, p = 0.011) in SOT compared to DOT, but at 6 weeks, the difference was not statistically significant (69.5% vs. 59.2%, p = 0.124). Cox proportional hazards analysis identified DOT and Dacron graft use as independent predictors of early neoMHV thrombosis. With regards their clinical Outcomes SOT had slightly shorter graft implantation time (41 min vs. 49 min, p = 0.002). In-hospital mortality was lower in SOT (2.7% vs. 9.2%, p = 0.044), but no difference in 1-year survival. NeoMHV thrombosis before 4 weeks was associated with worse morbidity and early mortality, underscoring the importance of early outflow patency. Overall this is a good quality study on a very specialised procedure within LDLT, they constructed a multicentre RCT design with reasonably balanced groups. They recognise the potential limitations of potential centre-specific protocol variations and short-to-medium follow-up for patency. In right lobe LDLT requiring anterior sector venous reconstruction, single outflow technique in the correct hands appears to achieve better early venous patency and may confer a survival advantage during the initial postoperative period. Further long-term data are required to evaluate late outcomes.

## Jadad Score

3.

## Data Analysis

Strict intention-to-treat analysis.

## Allocation Concealment

Yes.

## Trial Registration

CTRI Number - REF/2021/08/046152.

## Funding Source

No funding received.

**Randomised Controlled Trial 2**

Extracorporeal Photopheresis for the prevention of rejection after lung transplantation - a prospective randomized controlled trial.

*by Benazzo, A., et al. European Respiratory Journal 2024 [record in progress].*

## Aims

This study aimed to examine whether extracorporeal photopheresis (ECP) was effective as a prophylactic treatment for preventing acute cellular rejection (ACR), incidence of (CMV) infections as well as for reducing the risk of chronic lung allograft dysfunction (CLAD), in lung transplant recipients.

## Interventions

Participants were randomly assigned to receive either ECP plus standard triple-drug immunosuppression or standard triple-drug immunosuppressive treatment alone.

## Participants

31 lung transplant recipients.

## Outcomes

The primary outcome was a composite of high-grade ACR, CMV infection or CLAD. The secondary outcomes included ACR and lymphocytic bronchiolitis frequency, patient survival, graft survival, immune cell phenotyping, detection of plasma CMV DNA, number of antibody-mediated rejection (AMR) episodes, use of antilymphocyte globulin, and the incidence of clinically treated infections, *de novo* donor specific antibodies (dnDSAs), CLAD and serious adverse events (SAE).

## Follow-Up

24 months.

## CET Conclusion

*by John O’Callaghan*

This is a well-written report of a very interesting study in lung transplantation. The results are significant, showing a considerable and statistically significant reduction in acute rejection when extracorproeal photophoresis (ECP) was used in addition to standard immune suppression. This treatment was also associated with a significant reduction in infectious complications and chronic lung allograft dysfunction at 24 months, and lower hospital admissions. The group allocation could not be blinded, due to the nature of the ECP treatment, but the primary outcome is robust and the randomisation method reliable. Over 77% of patients in the ECP group received 90% of ECP treatment sessions. There was no significant difference in patient survival, however the study is likely to be underpowered for that outcome. The study only included patients transplanted for COPD, affecting generalizability to other indications for lung transplant.

## Jadad Score

3.

## Data Analysis

Strict intention-to-treat analysis.

## Allocation Concealment

Yes.

## Trial Registration

ClinicalTrials.gov - NCT05721079.

## Funding Source

Industry funded.

## Clinical Impact summary

*by John O’Callaghan*

This paper reports on a study that has the potential to influence clinical treatment protocols. The trial was conducted in clinical lung transplantation for COPD. Extracorporeal photopheresis was incorporated into a standard immune suppression regime and the primary outcome of interest was a composite outcome defined as incidence of high-grade acute cellular rejection, CMV infection or chronic lung-allograft dysfunction within 24 months after transplantation. The photopheresis system requires 1,500 mL of the patient’s blood, separated to isolate white blood cells, which are treated with a photosensitizing agent (like methoxsalen) and exposed to ultraviolet light. The modified “immunomodulated” white blood cells are then reinfused back into the patient. All patients received PCP and CMV prophylaxis as well as a protocol bronchoscopy with bronchiolar lavage and transbronchial biopsy at weeks 2, 4, 8, 12, 24 and 52. Additional bronchoscopies were performed if clinically indicated.

The treatment was associated with a significant reduction in the primary outcome: Freedom from the primary composite endpoint was 93% at 1 year and 76% at 2 years (compared to 52% and 45% in the control arm). The treatment was also associated with a significant reduction in high grade acute cellular rejection. It is very interesting that the intervention was associated with a reduction in rejection as well as a reduction in infections. There was a significantly higher incidence of SAEs in the control group, particularly infections, but not CMV.

The mechanism through which ECP modulates immune cell activity is not fully understood, and this study showed no shift in subpopulations between the control and study groups during the trial. This trial has shown some very promising results that warrant a multicentre study to follow-up.

### Clinical Impact

4/5.

